# Proactive Therapeutic Drug MONiToring to Guide Suppressive Antibiotic Therapy with DALBAvaNcin ( > 12 weeks) in Osteoarticular Infections (MONTALBANO)

**DOI:** 10.5194/jbji-10-255-2025

**Published:** 2025-07-30

**Authors:** Chiara Mariani, Matteo Passerini, Lucia Galli, Alice Covizzi, Marta Colaneri, Martina Offer, Margherita Faenzi, Stefania Merli, Simona Landonio, Marta Fusi, Alberto Dolci, Andrea Gori, Dario Cattaneo

**Affiliations:** 1 Department of Infectious diseases, Luigi Sacco Hospital, ASST Fatebenefratelli Sacco, via GB Grassi 74, Milan, Italy; 2 Department of Biomedical and Clinical Sciences (DIBIC), Università degli Studi di Milano, Via G.B. Grassi 74, 20157 Milan, Italy; 3 Department of Pathophysiology and Transplantation, Università degli Studi di Milano, via GB Grassi 74, Milan, Italy; 4 Unit of Clinical Pathology, ASST Fatebenefratelli Sacco University Hospital, Milan, Italy; 5 Centre for Multidisciplinary Research in Health Science (MACH), Università degli Studi di Milano, 20157 Milan, Italy

## Abstract

**Introduction**: Long-term dalbavancin use is increasingly adopted off-label for osteoarticular infections (OAIs), but data on administration timing and long-term effects beyond 12 weeks are scarce. This study evaluated the pharmacological efficacy of proactive therapeutic drug monitoring (TDM) to optimize dalbavancin administration. **Methods**: This single-center, retrospective study included adult OAI patients treated with ≥4 doses of dalbavancin from July 2022 to October 2024. Initial doses were given on days 1, 8, and 43. From the third dose onward, $C_{min}$ and $C_{max}$ values informed dosing schedules via log-linear regression models, targeting $C_{min}\geq 8$ mg L^−1^. The primary outcome was the pharmacological efficacy of dalbavancin, assessed by the proportion of patients with $C_{min}\geq 8$ mg L^−1^ and ≥4 mg L^−1^ after the third dose. Clinical outcomes and safety data were collected as descriptive data. **Results**: A total of 33 patients provided 118 $C_{min}$ determinations. Pharmacological efficacy was achieved in 93 / 118 (78.8 %) and 114 / 118 (96.6 %) determinations for $C_{min}$ thresholds of ≥8 mg L^−1^ and ≥4 mg L^−1^, respectively. Efficacy improved when considering only determinations at the correct timing. A total of 18 (54.5 %) patients are still in treatment, while 11 (33.3 %) completed therapy with clinical success. Three patients experienced a relapse after the end of the treatment, while one patient experienced failure, and no adverse events were reported. **Conclusions**: Dalbavancin is a viable option for prolonged OAI management when other therapies are unavailable or high-risk. Proactive TDM effectively supports this approach by ensuring adequate drug exposure while preventing accumulation.

## 1 Introduction

Dalbavancin is a long-acting lipoglycopeptide derived from a teicoplanin-like antibiotic (Malabarba and Goldstein, 2005) with activity against gram-positive bacteria (Weber et al., 2021) (Mendes et al., 2016).

Dalbavancin is characterized by a long half-life (14.4 d) and a total binding protein of 93 % (Rappo et al., 2019a). It is primarily cleared through renal pathways, lacks active metabolites, and is unaffected by cytochrome P450 inhibitors or other enzyme-inducing drugs (Buckwalter and Dowell, 2005). It was approved by the Food and Drug administration (FDA) in 2014 and subsequently by the European Medicines Agency (EMA) in 2015 for the treatment of acute bacterial skin and skin structure infections (ABSSIs) in one dose of 1500 mg or in two doses of 1000 and 500 mg 1 week apart (Boucher et al., 2014; Dunne et al., 2016). The drug is generally well tolerated, with nausea, headache, diarrhoea, vomiting, and mild rash occurring as the most common adverse events (AEs) (Gatti et al., 2021), while severe AEs such as acute renal injury or anaphylaxis reactions occurred only rarely (Bork et al., 2019).

Dalbavancin is also characterized by a decent penetration proportion (13.1 %) into bone tissue (Dunne et al., 2015) and into staphylococcal biofilms (Silva et al., 2021), making this antibiotic a promising weapon for the treatment of osteoarticular and biofilm-mediated infections. Thus, dalbavancin showed increasing evidence of efficacy in off-label regimens for infections due to gram-positive multidrug-resistant (MDR) organisms requiring prolonged treatment such as osteoarticular infections (OAIs) (Rappo et al., 2019b; Esposito et al., 2024). Moreover, dalbavancin, with its extended dosing interval, represents a valuable alternative to daily IV antibiotics, particularly in cases where oral therapy is either unfeasible or contraindicated (Azamgarhi et al., 2025). Dalbavancin may also be suitable for clinical scenarios requiring suppressive antibiotic therapy (SAT), a practice that still lacks clear guidelines for optimal management (Horne et al., 2024).

Despite its increasing use, the timing and dosage for off-label and long-term administration of dalbavancin remain undefined. Recent evidence supports the use of therapeutic drug monitoring (TDM) to estimate the duration of effective treatment in patients with OAIs (Cojutti et al., 2021). In addition, a recent expert opinion proposed regimens to achieve adequate dalbavancin concentration of up to 12 weeks (Senneville et al., 2023). Nonetheless, data on treatment durations beyond this period are still limited, and no specific recommendations are currently available regarding administration schedules or potential long-term effects.

At our center, as observed in other settings (Wouthuyzen-Bakker et al., 2017; Hanssen et al., 2024), we identified a subgroup of patients with OAIs, particularly hardware-associated infections, who require treatment exceeding 12 weeks due to a high risk of failure with shorter regimens and ineligibility for revision surgery. To address this, we developed a TDM-based approach to proactively guide dalbavancin administration in treatments lasting longer than 12 weeks (Cattaneo et al., 2024). Thus, the aim of this study is to assess the pharmacological efficacy of our method for OAIs and, secondly, to evaluate its clinical effectiveness and safety. Building on our previous data (Cattaneo et al., 2023, 2024), this study focuses exclusively on TDM observations relevant to extending treatment beyond 12 weeks, specifically for OAIs. It also expands the patient cohort, including clinical data on baseline characteristics, efficacy, and safety.

## 2 Methods

### 2.1 Study design and setting

We conducted a single-center observational study from the registry of off-label drugs considering patients with OAIs treated with dalbavancin at Luigi Sacco Hospital, in the Outpatients Parenteral Antimicrobial Therapy (OPAT) clinic, from July 2022 to October 2024. After planning the study and the research questions, we retrospectively collected data.

### 2.2 Participants

We included all adult patients ( >18 years) who received at least four administrations of dalbavancin for OAIs and for whom TDM was performed starting from at least the fourth dose onward. We included only cases with at least four administrations of dalbavancin because the first three doses are administered at predefined time points, whereas the fourth dose is the first one adjusted based on the pharmacological method applied at our center (see Sect. 2.3). We excluded pediatric patients, patients receiving fewer than four dalbavancin administrations, patients without information about estimated data for the fourth dalbavancin administration, patients without minimum plasma concentrations ( $C_{min}$ ) at the fourth dose, patients with indications other than OAIs, and patients without informed consent.

### 2.3 Timing of dalbavancin administrations

**Figure 1:**
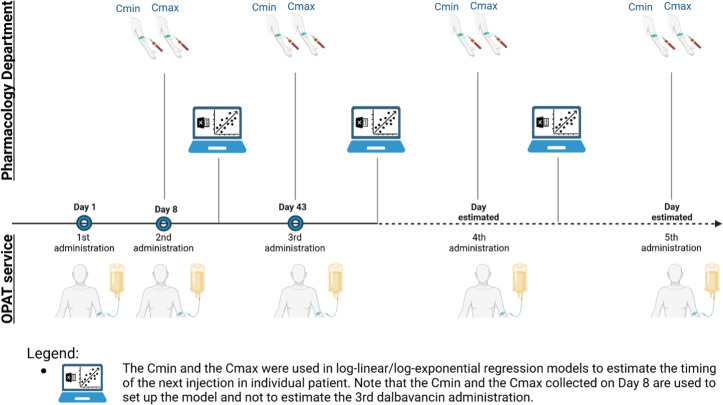
Clinical practice in OPAT service applying a TDM-based method to plan dalbavancin administrations for a duration of treatment longer than 12 weeks. Created in https://BioRender.com (last access: 5 May 2025).

Included patients received the first three dalbavancin doses on day 1 (T0), day 8 (T1), and day 43 (T2) (Senneville et al., 2023). Subsequent injection timings were personalized and determined using dalbavancin $C_{min}$ measured immediately before the dalbavancin administration and maximum plasma concentrations ( $C_{max}$ ) measured 30 min after the end of the injection (Fig. 1). Starting from the third dose, $C_{min}$ and $C_{max}$ values were used in log-linear regression models by plotting logarithmic dalbavancin concentrations with time (Microsoft Excel Software, Microsoft Company, USA) (Cattaneo et al., 2023) to plan the subsequent administration aiming to maintain $C_{min}$ levels at ≥8 mg L^−1^, as recommended in the recent literature (Cojutti et al., 2021).

### 2.4 Assessment of plasma dalbavancin concentrations

Plasma dalbavancin blood samples were assessed using ethylene–diamine–tetraacetic acid (EDTA)-containing Vacutainers^®^. All samples were centrifuged at 3000 × g and plasma was separated and stored at -20 °C. Dalbavancin quantification was performed through a validated liquid chromatography tandem mass spectrometry method developed and validated according to the EMA guidelines. The analytical process consisted of a fast protein precipitation protocol of 50 µ L of plasma with 400 µ L of precipitation solution (methanol / acetonitrile, 3 / 1), centrifugation at 10 000 × g , 1 : 3 dilutions with water, and analysis. Chromatographic separation was achieved using a gradient (acetonitrile and water with formic acid 0.1 %) on a reversed-phase analytical column (Acquity UPLC BEH C18 1.70 µ m 2.1 × 50 mm; Waters, Milan, Italy). For quantification, analysis was performed in ESI-positive mode by monitoring the transition m/z = 909.45 > 340.2 for dalbavancin. The method was linear from 1 to 500 mg L^−1^, with intraday and interday assay imprecision and inaccuracy consistently < 10 % during each analytical run.

### 2.5 Primary outcome

The primary outcome was to evaluate the pharmacological efficacy of our proactive TDM-based administration method in maintaining the dalbavancin $C_{min}$ level at ≥8 mg L^−1^ or ≥4 mg L^−1^ taken from the fourth dose onward.

### 2.6 Other descriptive data of interest

We also collected clinical effectiveness and safety data. Given the observational nature of the study and the limited sample size, these outcomes were collected as descriptive data.

### 2.7 Definitions

#### 2.7.1 Pharmacological efficacy

Pharmacological efficacy was defined as the number of instances where $C_{min}\geq 8$ mg L^−1^(or ≥4 mg L^−1^) was achieved from the fourth dose onward. The thresholds of 4 and 8 mg L^−1^ were selected based on prior literature, which suggests that these concentrations ensure, with approximately 90 % probability, the attainment of pharmacodynamic targets against *Staphylococcus aureus* for MIC values up to MIC_90_ (0.0625 mg L^−1^) and the EUCAST clinical susceptibility break point for dalbavancin (0.125 mg L^−1^), respectively (Dunne et al., 2015; Cojutti et al., 2021). A tolerance window of up to ±4 d from the calculated injection date was allowed to adapt the model to real-life conditions and improve adherence to the treatment regimen.

#### 2.7.2 Clinical effectiveness

We established effectiveness with an assessment of clinical and microbiological outcomes from chart review detected up to 31 October 2024. If the patient was still on dalbavancin treatment, the clinical outcome was assessed as “ongoing treatment”. Otherwise, the assessment could result in the following: (i) completed therapy with clinical success (absence of failure or relapse at the follow-up); (ii) failure (clinical or microbiological failure with uncontrolled infection during dalbavancin treatment; need for medical interventions for the treated episode in addition to the initial program); (iii) relapse (exacerbation of the clinical condition within 6 months after the end of treatment); (iv) discontinuation of therapy due to AEs; (v) discontinuation of therapy due to new hospitalization for other clinical events; (vi) missing data (insufficient details to assess the outcome; lost at follow-up); (vii) death for infection-related conditions; or (viii) death for non-infection-related conditions.

#### 2.7.3 Safety

Safety was assessed by monitoring AEs. We defined renal toxicity as a reduction in the estimated glomerular filtration rate (eGFR) to less than 30 mL min^−1^/1.73 m^2^, accompanied by a decrease of at least 20 % from baseline. A significant reduction in platelet count was considered when the count fell below 100 000 per mm^−3^ with a decrease of at least 25 % from baseline.

### 2.8 Data source and extraction

We identified the patients from the registry of off-label drugs and the registry of the OPAT clinic, which collects all the individuals receiving dalbavancin in an off-label regimen. Then we collected data from electronic records using “NOEMALIFE Galileo” software and manual chart review from the OPAT clinic. Extracted data included age, sex, body mass index (BMI), comorbidities, type of OAI, presence of hardware, isolated pathogens, concomitant antibiotics, source control, number of doses and dosage of dalbavancin, duration of dalbavancin therapy, information about the fourth dalbavancin administration and the subsequent administrations (date estimated by log-linear regression method for antibiotic administration, date effective of antibiotic administration, dalbavancin $C_{min}$ concentration), laboratory tests (serum albumin at T0; platelet count, serum creatinine, and eGFR at baseline (T0) and at 7±3 d of therapy (T1), at 6 weeks ± 10 d of therapy (T2), at 12 weeks ± 10 d of therapy (T3), at 6 months of therapy (T4), at 12 months of therapy (T5) and 18 months of therapy (T6)), potentially related AEs (significant platelet count reduction, acute renal failure, dermatological manifestation, angioedema, and other), clinical outcome, and duration of follow-up. Data were recorded in Microsoft Excel v16.0.

### 2.9 Statistical methods

To determine the timing of dalbavancin administration, refer to Sect. 2.3. To describe the baseline characteristics and the outcomes, we described continuous variables as a median (interquartile range – IQR), while categorical variables are described as counts and percentages of positive responses.

## 3 Results

### 3.1 Participants

**Figure 2:**
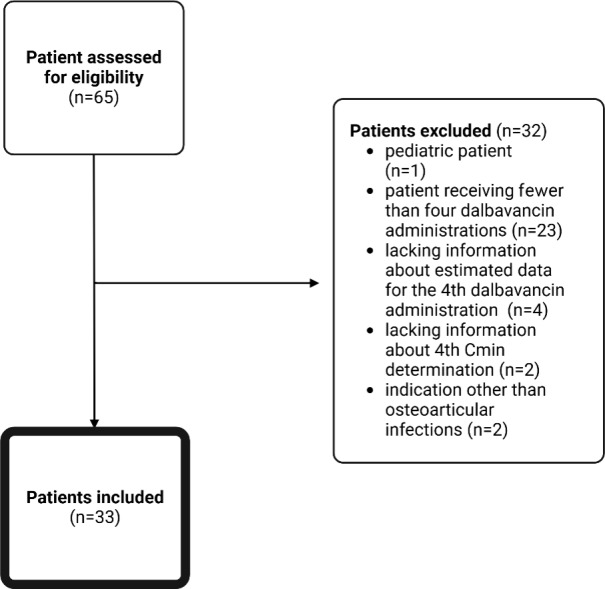
Patient inclusion flowchart.

The registries of the off-label drugs and OPAT clinic identified 65 patients. The final analysis included 33 patients (Fig. 2).

### 3.2 Descriptive data

**Figure 3:**
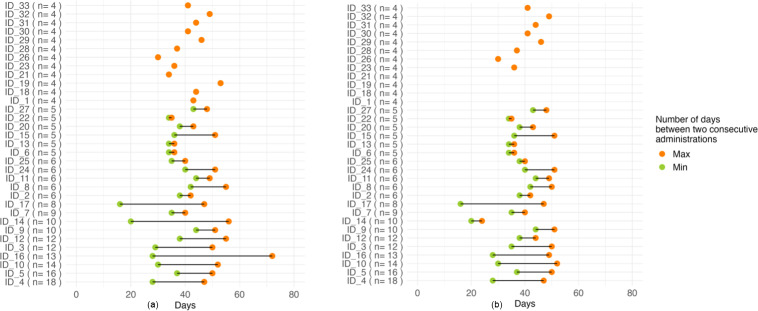
The left panel represents the interval between dalbavancin administrations in days (after excluding the first three doses). The number in parentheses indicates the number of dalbavancin administrations received by each patient. The right panel represents the same interval after excluding the administrations beyond 7 d from the suggested date (note the difference in patients ID_14 and ID_16).

Most of the included patients were males (20 / 33, 60.6 %); median age was 73 years (interquartile range, IQR: 58–78) and median BMI was 25.7 (IQR, 23.7–27.7), identifying a median overweight population. A total of 25 out of 33 patients (75.8 %) had hardware-associated bone and joint infections. Median (range, minimum to maximum) baseline serum albumin, creatinine, and eGFR were 36 (25 to 47) g L^−1^, 0.97 (0.45 to 2.07) mg dL^−1^, and 81.25 (17.2 to 214.1) mL min^−1^/1.73 m^2^, respectively. Patients received a median of 5 (range, 4–18) injections over a median period of 133 d (IQR, 87–226) between the first and the last dalbavancin administration. All patients received 1500 mg of dalbavancin as a first dose; 31 / 33 (93.9 %) continue with this dosage, while 2 / 33 (6.1 %) patients received 1000 mg in a maintenance dose due to their chronic kidney disease (baseline eGFR was 35.2 and 17.2 mL min^−1^/1.73 m^2^, respectively). Additional patient demographic and clinical characteristics are summarized in Table 1. We observed substantial variability in administration intervals across patients, whereas intra-patient variability remained relatively limited and was further reduced upon exclusion of administrations occurring more than 7 d beyond the recommended time frame (Fig. 3).

**Table 1:** Baseline characteristics of the study population.

	Patients ( n=33 )
Age years, median (IQR)	73 (58–78)
Male, n (%)	20 (60.6)
BMI, median (IQR)	25.7 (23.7–27.7)
Comorbidities, n (%) Diabetes Cardiovascular disease Chronic pulmonary disease Chronic renal disease Chronic liver disease HIV infection Immunosuppression Ongoing oncologic condition	n = 42 7 (21.2) 22 (66.6) 5 (15.6) 4 (12.1) 1 (3) 1 (3) 1 (3) 1 (3)
Types of infections, n (%) PJI NJA NVO Hardware-associated vertebral osteomyelitis Native non-vertebral osteomyelitis Hardware-associated non-vertebral osteomyelitis	n = 34 16 (48.5) 1 (3) 0 3 (9.1) 6 (18.2) 8 (24.2)
Presence of hardware, n (%)	25 (75.8)
Polymicrobial infection, n (%)^a^	11 (33.3)
Isolated pathogens, n (%)^b^ MSSA MRSA CoNs MS CoNs MR *E. faecalis.* *Streptococcus* spp.	n = 44 12 (27.3) 11 (25) 9 (20.5) 5 (11.4) 4 (9.1) 3 (6.8)
Interval (days) between the first and the last dalbavancin administration, median (IQR)	133 (87–226)
Number of dalbavancin administrations, median (range)	5 (4–18)
Source control, n (%)	23 (69.7)
Concomitant antibiotic therapy, n (%)	2 (6.1)

The abbreviations used in the table are as follows. IQR: interquartile range; BMI: body mass index; HIV: human immunodeficiency virus; NJA: native joint arthritis; NVO: native vertebral osteomyelitis; MSSA: methicillin-sensitive *Staphylococcus aureus*; MRSA: methicillin-resistant *Staphylococcus aureus*; CoNS MS: methicillin-sensitive coagulase-negative staphylococcus; CoNS MR: methicillin-resistant coagulase-negative staphylococcus. ^a^ 10 / 11 due to gram-positive pathogens, 1 / 11 due to gram-negative pathogens. ^b^ MIC for vancomycin for 19 isolates: 2 / 19 MIC < 1, 13 / 19 MIC = 1, 2 / 19 MIC = 2; we calculated MIC for dalbavancin just for one patient and it resulted in < 0.06.

### 3.3 Primary outcome

A total of 93 of 118 (78.8 %) and 114 / 118 (96.6 %) $C_{min}$ resulted in ≥8 mg L^−1^ and ≥4 mg L^−1^, respectively. A total of 95 of 118 (80.5 %) through concentrations ( $C_{\min}$ ) were performed in the time window suggested by the model; among these 81 / 95 (85.3 %) were ≥8 mg L^−1^ and 93 / 95 (97.9 %) were ≥4 mg L^−1^ (Table 2).

**Table 2:** Pharmacological efficacy of the TDM-based timing method (see Sect. 2.6).

	Total $C_{min}$ ( n=118 )	$C_{min}$ performed at correct timing ( n=95 )
$C_{min}\geq 8$ mg L^−1^, n (%)	93 (78.8)	81 (85.3)
$C_{min}\geq 4$ mg L^−1^, n (%)	114 (96.6)	93 (97.9)

The abbreviations used in the table are as follows. TDM: therapeutic drug monitoring; $C_{min}$ : dalbavancin minimum plasma concentration.

### 3.4 Secondary outcomes

A total of 18 out of 33 (54.5 %) patients were still undergoing treatment with controlled infection at the end of the study, and 11 / 33 (33.3 %) completed therapy with clinical success. Three patients experienced a relapse of osteomyelitis: one at the level of the fifth metatarsal 6 months after completing therapy and two others approximately 2 months post-therapy, involving the ankle and leg, respectively, in regions where hardware remained in situ. Finally, one patient had to discontinue dalbavancin due to a new hospitalization for a new infection, possibly related to the original focus; we considered it a failure. All four patients with relapse or failure had at least one $C_{min}<8$ mg L^−1^, and 1/4 had at least one value <4 mg L^−1^. Among the 29 patients with clinical success, 15 had at least one $C_{min}<8$ mg L^−1^ and 12 had at least one <4 mg L^−1^.

None of the 33 included patients experienced AEs (Table 3).

**Table 3:** Clinical outcomes of the included patients.

	Patients ( n=33 )
Ongoing, n (%)	18 (54.5)
Completed therapy with clinical success, n (%)	11 (33.3)
Failure, n (%)	1 (3)
Relapse, n (%)	3 (9.1)
Time of FU, median (IQR)	79.5 (65–182)
AEs, n (%)	0

The abbreviations used in the table are as follows. AEs: adverse events; FU: follow-up.

## 4 Discussion

Our study collected data from a cohort of patients with OAIs treated with dalbavancin for over 12 weeks, utilizing a proactive TDM-based approach to evaluate its pharmacological efficacy, clinical effectiveness, and safety. Pharmacological efficacy was achieved in more than three-quarters of the study population based on the concentration targets reported in the literature for OAIs caused by *Staphylococcus aureus*. Furthermore, most of the study population either continued treatment or achieved clinical success without AEs, suggesting the clinical effectiveness and safety of our TDM-based approach.

Currently, there is considerable heterogeneity in the use of SAT with dalbavancin for OAIs in both the initial and the maintenance doses. For the latter, some proposed regimens range from 500 mg weekly to 500 mg biweekly to 1000 mg biweekly (Martín et al., 2019; Morata et al., 2019; Wunsch et al., 2019). However, none of these studies employed TDM to assess the pharmacological efficacy of such regimens. Söderquist et al. (2024) recently shared their experience with 12 patients treated with long-term ( >12 weeks) dalbavancin for PJI, according to the Swedish national guidelines and introducing TDM. These patients received 1500 mg on day 1, day 8, and day 14, followed by 1000 mg biweekly starting on day 28. Prospective sampling for dalbavancin $C_{min}$ was conducted biweekly. All resulting $C_{min}$ values were well above the therapeutic target of 8 mg L^−1^, and three patients showed a tendency towards progressive accumulation of dalbavancin during treatment (Söderquist et al., 2024).

In this context, our study introduces a novel TDM-based approach to manage long-term dalbavancin treatment for OAIs requiring more than 12 weeks of therapy. Most of the $C_{min}$ values obtained with our method remained above the therapeutic target without leading to drug accumulation. Notably, our results improved when we considered only $C_{min}$ measurements collected within in the time window recommended by our method. Our approach, in most cases, ensures adequate drug levels and prevents sub-therapeutic serum concentrations, thereby potentially reducing the risk of failure and development of antibiotic resistance. Secondly, this proactive approach enables the prevention of dalbavancin accumulation rather than requiring dose adjustments after accumulation has already occurred. Specifically, we did not observe any adverse effects on renal function or platelet count, which could have been expected given the known potential adverse effects of glycopeptide-derived drugs.

Our TDM approach is hypothesized to have a positive impact not only on efficacy and safety but also on resource management. Administering the correct dose to patients while avoiding unnecessary additional doses can lead to significant cost savings, particularly given the relatively low cost of a single TDM assay or the use of a clinical pharmacology reference center serving as a hub for multiple facilities. Furthermore, since dalbavancin's $C_{min}$ and $C_{max}$ measurements, as well as its administration, are conducted within the same half-day, this strategy also saves time and resources for both patients and healthcare staff. TDM was performed when the patient came for the next dose, which suits OPAT logistics. However, based on our observations, intra-patient variability was higher in the first four to five doses and tended to stabilize later. Thus, TDM might be more critical early in treatment and less frequent thereafter.

Our study has several limitations. First, its retrospective design results in significant missing data, such as minimum inhibitory concentration (MIC) values for dalbavancin and variability in follow-up duration, which could potentially affect the accuracy of clinical outcome data related to effectiveness. Second, we included a heterogeneous and small population, and we did not use a comparator group without TDM-based approach. This does not allow us to assess effectiveness and safety with strong evidence. However, the primary outcome of our study was to assess the pharmacological efficacy of our approach. Third, the choice of 8 and 4 mg L^−1^ as $C_{min}$ targets is based on studies conducted on *Staphylococcus aureus*, rather than on our study population, which also included OAIs caused by other pathogens, such as *Enterococcus faecalis* and *Streptococcus* spp. The 8 mg L^−1^ target derives from literature on protein binding, bone penetration, and EUCAST MICs for *S. aureus*. However, the optimal target likely varies with the pathogen's MIC and infection site. We also suspect serum albumin may affect the active drug fraction, but further data are needed. Fourth, there were unexpected events related to clinical practice. Specifically, from 15 February to 24 April 2024, our Pharmacology Department was unable to perform dalbavancin TDM due to machinery failure, and therefore the appointments were based on previous calculated treatment intervals. Additionally, in May 2024, insufficient drug availability led to one patient receiving oral therapy with trimethoprim–sulfamethoxazole for 1 month before resuming dalbavancin. During the same period, one patient previously considered for suppressive antibiotic therapy interrupted treatment from 4 to 30 May 2024 before restarting dalbavancin infusions. Lastly, we measured total concentration rather than free concentration, which is thought to be more reliable, since free concentration–time profiles may best correlate with therapeutic effect. Nevertheless, measuring free concentrations is technically challenging, resource-intensive, and not yet feasible for routine clinical practice.

We also acknowledge two factors that currently limit the external applicability of this method. The first is that not all centers are equipped with a clinical pharmacology service capable of performing TDM and supporting patient care. The second is that prolonged use of dalbavancin may be economically unsustainable for some centers. The first limitation can be mitigated, as previously mentioned, by establishing a center that acts as a regional hub for other facilities. Additionally, one of the future goals of our working group is to develop a model that can be utilized even when TDM values are not available. Although preliminary analyses suggested that variables such as BMI may influence dosing intervals, the limited dataset in this study rendered the statistical analyses unreliable. We plan to expand the cohort to better investigate these correlations and potentially incorporate them into the proposed model. The second limitation necessitates careful patient selection to identify those who are most likely to benefit from dalbavancin use. For instance, as has been recently emphasized, every effort should be made to switch from IV to oral therapy whenever feasible and when the expected benefits outweigh the risks, considering the individual patient's circumstances and antimicrobial stewardship practices (Senneville et al., 2023). Furthermore, we believe that collaboration with national regulatory authorities is essential to facilitate easier access to the drug, even for off-label use, in cases where other therapeutic options are unavailable.

Despite these limitations, our study highlights the potential role of dalbavancin in long-term treatment. This role could be further enhanced by a TDM-based approach, which enables more precise timing of dalbavancin administration. Such guidance ensures adequate drug concentrations, minimizes the risks of accumulation and associated adverse events, prevents sub-therapeutic serum levels that could reduce effectiveness, and avoids unnecessary economic waste.

## Data Availability

The code and data used in this work are available from the corresponding author upon request.

## References

[bib1.bib1] Azamgarhi T, Warren S, Scobie A, Karunaharan N, Perez-Sanchez C, Houghton R, Hassan S, Lourtet-Hascoët J, Kershaw H, Sendi P, Saeed K (2025). Dalbavancin to facilitate early discharge in the treatment of complex musculoskeletal infections: a multi-centre real-life application. J Bone Joint Infect.

[bib1.bib2] Bork JT, Heil EL, Berry S, Lopes E, Davé R, Gilliam BL, Amoroso A (2019). Dalbavancin Use in Vulnerable Patients Receiving Outpatient Parenteral Antibiotic Therapy for Invasive Gram-Positive Infections. Infect Dis Ther.

[bib1.bib3] Boucher HW, Wilcox M, Talbot GH, Puttagunta S, Das AF, Dunne MW (2014). Once-Weekly Dalbavancin versus Daily Conventional Therapy for Skin Infection. New Engl J Med.

[bib1.bib4] Buckwalter M, Dowell JA (2005). Population Pharmacokinetic Analysis of Dalbavancin, a Novel Lipoglycopeptide. J Clin Pharmacol.

[bib1.bib5] Cattaneo D, Fusi M, Colaneri M, Fusetti C, Genovese C, Giorgi R, Matone M, Merli S, Petri F, Gori A (2023). Therapeutic Drug Monitoring of Dalbavancin in Real Life: A Two-Year Experience. Antibiotics.

[bib1.bib6] Cattaneo D, Fusi M, Galli L, Genovese C, Giorgi R, Matone M, Merli S, Colaneri M, Gori A (2024). Proactive therapeutic monitoring of dalbavancin concentrations in the long-term management of chronic osteoarticular/ periprosthetic joint infections. Antimicrob Agents Ch.

[bib1.bib7] Cojutti PG, Rinaldi M, Gatti M, Tedeschi S, Viale P, Pea F (2021). Usefulness of therapeutic drug monitoring in estimating the duration of dalbavancin optimal target attainment in staphylococcal osteoarticular infections: a proof-of-concept. Int J Antimicrob Agents.

[bib1.bib8] Dunne MW, Puttagunta S, Sprenger CR, Rubino C, Van Wart S, Baldassarre J (2015). Extended-duration dosing and distribution of dalbavancin into bone and articular tissue. Antimicrob Agents Ch.

[bib1.bib9] Dunne MW, Puttagunta S, Giordano P, Krievins D, Zelasky M, Baldassarre J (2016). A Randomized Clinical Trial of Single-Dose Versus Weekly Dalbavancin for Treatment of Acute Bacterial Skin and Skin Structure Infection. Clin Infect Dis.

[bib1.bib10] Esposito S, Pagliano P, De Simone G, Guarino A, Pan A, Brambilla P, Mastroianni C, Lichtner M, Brugnaro P, Carretta A, Santantonio T, Brindicci G, Carrega G, Montagnani F, Lapadula G, Spolti A, Luzzati R, Schiaroli E, Scaglione V, Pallotto C, Tacconi D, Quintieri F, Trecarichi E (2024). In-label, off-label prescription, efficacy and tolerability of dalbavancin: report from a National Registry. Infection.

[bib1.bib11] Gatti M, Andreoni M, Pea F, Viale P (2021). Real-World Use of Dalbavancin in the Era of Empowerment of Outpatient Antimicrobial Treatment: A Careful Appraisal Beyond Approved Indications Focusing on Unmet Clinical Needs. Drug Des Devel Ther.

[bib1.bib12] Hanssen JLJ, van der Wal RJP, van der Linden HMJ, van Prehn J, Scheper H, de Boer MGJ (2024). Dosing and treatment duration of suppressive antimicrobial therapy in orthopedic implant infections: a cohort study. J Bone Jt Infect.

[bib1.bib13] Horne M, Woolley I, Lau JSY (2024). The Use of Long-term Antibiotics for Suppression of Bacterial Infections. Clin Infect Dis.

[bib1.bib14] Malabarba A, Goldstein BP (2005). Origin, structure, and activity in vitro and in vivo of dalbavancin. J Antimicrob Chemoth.

[bib1.bib15] Martín LB, Fernández MM, Ruiz JMP, Lafont MO, Paredes LÁ, Rodríguez MÁM, Regueras MF, Morón MÁM, Lobón GM (2019). Dalbavancin for treating prosthetic joint infections caused by Gram-positive bacteria: A proposal for a low dose strategy. A retrospective cohort study. Revista Espanola de Quimioterapia.

[bib1.bib16] Mendes RE, Castanheira M, Farrell DJ, Flamm RK, Sader HS, Jones RN (2016). Update on dalbavancin activity tested against Gram-positive clinical isolates responsible for documented skin and skin-structure infections in US and European hospitals (2011–13): Table 1. J Antimicrob Chemoth.

[bib1.bib17] Morata L, Cobo J, Fernández-Sampedro M, Guisado Vasco P, Ruano E, Lora-Tamayo J, Sánchez Somolinos M, González Ruano P, Rico Nieto A, Arnaiz A, Estébanez Muñoz M, Jiménez-Mejías ME, Lozano Serrano AB, Múñez E, Rodriguez-Pardo D, Argelich R, Arroyo A, Barbero JM, Cuadra F, Del Arco A, Del Toro MD, Guio L, Jimenez-Beatty D, Lois N, Martin O, Martínez Alvarez RM, Martinez-Marcos FJ, Porras L, Ramírez M, Vergas García J, Soriano A (2019). Safety and efficacy of prolonged use of dalbavancin in bone and joint infections. Antimicrob Agents Ch.

[bib1.bib18] Rappo U, Puttagunta S, Shevchenko V, Shevchenko A, Jandourek A, Gonzalez PL, Suen A, Mas Casullo V, Melnick D, Miceli R, Kovacevic M, De Bock G, Dunne MW (2019). Dalbavancin for the Treatment of Osteomyelitis in Adult Patients: A Randomized Clinical Trial of Efficacy and Safety. Open Forum Infect Dis.

[bib1.bib19] Rappo U, Dunne MW, Puttagunta S, Baldassarre JS, Su S, Desai-Krieger D, Inoue M (2019). Epithelial Lining Fluid and Plasma Concentrations of Dalbavancin in Healthy Adults after a Single 1,500-Milligram Infusion. Antimicrob Agents Ch.

[bib1.bib20] Senneville E, Cuervo G, Gregoire M, Hidalgo-Tenorio C, Jehl F, Miro JM, Seaton A, Söderquist B, Soriano A, Thalhammer F, Pea F (2023). Expert Opinion on Dose Regimen and Therapeutic Drug Monitoring for Long-Term Use of Dalbavancin: Expert Review Panel. Int J Antimicrob Agents.

[bib1.bib21] Silva V, Miranda C, Bezerra M, Antão HS, Guimarães J, Prada J, Pires I, Maltez L, Pereira JE, Capelo JL, Igrejas G, Poeta P (2021). Anti-biofilm activity of dalbavancin against methicillin-resistant *Staphylococcus aureus* (MRSA) isolated from human bone infection. J Chemotherapy.

[bib1.bib22] Söderquist B (2024). Trough levels of dalbavancin during long-term treatment of prosthetic joint infections.

[bib1.bib23] Weber RE, Fleige C, Layer F, Neumann B, Kresken M, Werner G (2021). Determination of a Tentative Epidemiological Cut-Off Value (ECOFF) for Dalbavancin and Enterococcus faecium. Antibiotics.

[bib1.bib24] Wouthuyzen-Bakker M, Nijman JM, Kampinga GA, Assen Sv, Jutte PC (2017). Efficacy of Antibiotic Suppressive Therapy in Patients with a Prosthetic Joint Infection. J Bone Joint Infect.

[bib1.bib25] Wunsch S, Krause R, Valentin T, Prattes J, Janata O, Lenger A, Bellmann-Weiler R, Weiss G, Zollner-Schwetz I (2019). Multicenter clinical experience of real life Dalbavancin use in gram-positive infections. Int J Infect Dis.

